# Association between NAFLD and risk of prevalent chronic kidney disease: why there is a difference between east and west?

**DOI:** 10.1186/s12876-020-01278-z

**Published:** 2020-05-06

**Authors:** Min Zhang, Su Lin, Ming-fang Wang, Jiao-feng Huang, Shi-ying Liu, Su-mei Wu, Hao-yang Zhang, Zi-mu Wu, Wen-Yue Liu, Dong-Chu Zhang, Chuan-ming Hao, Yue-yong Zhu, Ming-Hua Zheng, Xiao-zhong Wang

**Affiliations:** 1 Division of Nephrology, Huashan Hospital, Fudan University, 12 Middle Wulumuqi Road, Shanghai, China; 2 Department of Gastroenterology, Union Hospital of Fujian Medical University, Fuzhou, Fujian China; 3 Liver Research Center, the First Affiliated Hospital of Fujian Medical University, No. 20, Chazhong Road, Taijiang District, Fuzhou, Fujian China; 4 School of Biomedical Engineering, Sun Yat-sen University, Guangzhou, China; 5 Department of Neurology, Peking University Shenzhen Hospital, Shenzhen, China; 6 Department of Endocrinology, the First Affiliated Hospital of Wenzhou Medical University, Wenzhou, China; 7 Wenzhou Medical Center, Wenzhou People’s Hospital, Wenzhou, China; 8 NAFLD Research Center, Department of Hepatology, the First Affiliated Hospital of Wenzhou Medical University, Wenzhou, China; 9 Institute of Hepatology, Wenzhou Medical University, Wenzhou, China; 10 Key Laboratory of Diagnosis and Treatment for The Development of Chronic Liver Disease in Zhejiang Province, Wenzhou, China

**Keywords:** Non-alcoholic fatty liver disease, Chronic renal disease

## Abstract

**Backgrounds:**

There is a discrepancy between west and east on the relationship between non-alcoholic fatty liver disease (NAFLD) and chronic kidney disease (CKD). This study aimed to find out the possible reason for this and to clarify the association between NAFLD and CKD by analyzing two population-based datasets from the US and China.

**Methods:**

Two health examination datasets from China and the US were used. CKD was defined as an estimated glomerular filtration rate (eGFR) < 60 ml/min/1.73m^2^ or and/or abnormal albuminuria and/or overt proteinuria. Binary logistic regression was used to examine the association between NAFLD and CKD.

**Results:**

A total of 60,965 participants were analyzed, including 11,844 from the US and 51,229 from China. The prevalence of NAFLD was 27.12% in the Chinese population and 36.08% in the US population (*p* < 0.001). The proportions of CKD and late stage CKD (stages 3–5) were higher in the US population than the Chinese one. NAFLD was independently associated with an increased risk of CKD in Chinese population, whereas in the US population, the NAFLD was not an independent risk factor of CKD. In subgroup analyses which excluded late stages CKD (stages 3–5), the risks of mild renal function decline became consistent: NAFLD was associated with early stages of CKD but not the late stages of CKD in both populations.

**Conclusion:**

NAFLD increased the risk of early stages of CKD in both Chinese and the US population. The conflicting results reported by previous studies might result from the different proportion of late stages of CKD.

## Backgrounds

Non-alcoholic fatty liver disease (NAFLD) is characterized by lipid accumulation in the liver with the absence of significant alcohol intake or other medical conditions that cause fatty liver. It is one of the most common forms of liver diseases with a global prevalence up to 30% in general population [1]. NAFLD is also a multisystem disease, affecting extra-hepatic organs such as kidneys [2, 3].

The relationship between NAFLD and chronic kidney disease (CKD) has attracted much attention recently. NAFLD and CKD may share common pathogenic mechanisms, such as insulin resistance, type 2 diabetes mellitus, hyperlipidemia and obesity, and thus may potentially share same therapeutic targets [4–7]. Growing evidence suggests that patients with NAFLD have a higher risk of CKD than non-NAFLD population [8–12]. NAFLD was responsible for a higher frequency of simultaneous liver and kidney transplantation than other liver diseases, and also a higher rate for renal retransplantation after simultaneous liver and kidney transplantation [13]. According to previous report, 1/3 of transplant patients who received a NAFLD donor liver might develop stage 3 CKD within 2 years after transplantation [14]. These studies highlight the significance of NAFLD as a risk for CKD. However, studies from different regions have drawn controversial conclusions on the relationship between NAFLD and CKD. For example, evidence from US population showed no associated between NAFLD and CKD after adjustment for components of the metabolic syndrome [15], while a study from Asia reported a strong independent relationship between ultrasonography-diagnosed NAFLD and CKD [16]. A latest meta-analysis also confirmed the ethnic difference between Asian and European population: the association between NAFLD and the risk of CKD is stronger in Asian population while insignificant in European population [9]. The possible reason for these contradictory conclusions might due to the heterogeneity of the study population and the different variables being adjusted in each study, yet this hypothesis has not been clearly elucidated.

In this study, we aimed to analyze the association between NAFLD and CKD in two nationally representative datasets from the US and China and try to elucidate the risk of CKD in NAFLD population from different countries.

## Methods

### Participants

This was a cross-sectional study. Two health examination datasets were analyzed in this study. The US dataset was retrieved from the Third American National Health and Nutrition Examination Survey (NHANES III), a nationally representative, cross-sectional study conducted by the National Center for Health Statistics of the United States from 1984 to 1994. The dataset of this study and further information are available at https://www.cdc.gov/nchs/nhanes/about_nhanes.htm. The Chinese health examination dataset was retrieved from Wenzhou Medical Center of Wenzhou People’s Hospital, China, from January 2010 to December 2010.

Exclusion criteria included the presence of any of the following: without ultrasonography results, with missing data, with hepatitis B or C, and participants with excessive alcohol intake. In Chinese datasets, it was defined as > 140 g/week for men and > 70 g/week for women. In US datasets, it was defined as two drinks a day. A drink means a 12-oz beer, a 4-oz glass of wine or an ounce of liquor, approximately 35 g alcohol.

According to the presence of fatty liver in ultrasonography, participants were divided into the NAFLD group and non-NAFLD group.

### Anthropometric and biochemical measurements

The diagnosis of hepatosteatosis was based on ultrasonography in both populations. Serum cholesterol, triglyceride, serum creatinine, and uric acid were obtained from the original datasets. Body mass index (BMI) was calculated as weight (in kilograms) divided by the square of the height (in meters). Mean arterial pressure (MAP) was calculated as MAP = (systolic pressure + 2 x diastolic pressure) /3.

Estimated glomerular filtration rate (eGFR) was calculated according to the 2009 CKD-EPI eGFR formula [17]: eGFR = 141 x min(SCr/κ, 1)^α^ x max(SCr /κ, 1) ^-1.209^ × 0.993^Age^ x [1.018 if female] x [1.159 if Black];κ = 0.7 (females) or 0.9 (males); α = − 0.329 (females) or − 0.411 (males), where Scr is serum creatinine concentration (in mg/dL) and age refers to age in years.

CKD was defined as either decreased eGFR (< 60 ml/min/1.73^2^) and/or abnormal albuminuria and/or overt proteinuria, in accordance with the Kidney Disease: Improving Global Outcome (KIDGO) 2012 Practice guideline for CKD [18].

CKD was classified into five stages based on the eGFR categories. Decline in GFR category represented CKD stage G1 to G5 (≥90 [G1], 60–89 [G2], 59–30 [G3], 15–29 [G4], < 15 [G5] ml/min/ 1.73 m^2^). The early stages of CKD were defined as CKD stage G1-G2, while the late stages of CKD were defined as stages 3–5.

### Statistical analysis

Continuous variables were represented as mean ± standard deviation and compared using the Student’s t-test. Categorical variables were expressed as counts (percentages) and compared using the Chi-squared test or the Fisher’s exact test when the samples were limited in number. Binary logistic regressions including two models (Model 1 adjusted for age, sex, BMI. Model 2 adjusted for age, sex, BMI, history of diabetes and history of hypertension) were employed to find the relationship between renal function decline and the presence of NAFLD after correcting for different confounding factors of renal function. All tests were two-sided, and a *p*-value < 0.05 were considered statistically significant. All analysis was conducted by SPSS version 23.0.

## Results

### Characteristics of participants

**Fig. 1:**
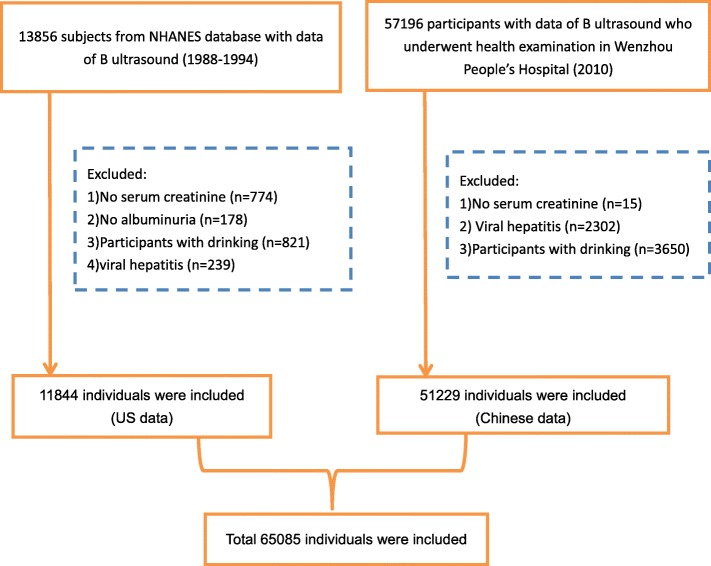
Flow-chart of cases screening

A total of 65,085 participants were included in this study (Fig. 1), with 11,844 from the NHANES III dataset (the US population) and 51,229 from the Chinese dataset (the Chinese population). The characteristics of all participants are shown in Tables 1 and 2. The difference in the age between two populations was statistically significant but less clinically significant (43.49 ± 14.30 vs. 43.86 ± 16.08, *p* = 0.023). Compared with the US population, the Chinese population was more likely to be male (59.70% vs. 39.48%, *p* = 0.019), had lower BMI levels (23.08 ± 3.31 vs. 27.47 ± 5.98, *p* < 0.001) and lower proportions of diabetes (6.9% vs. 7.6%, *p* < 0.001) or hypertension (21.8% vs. 25.5%, *p* < 0.001).

**Table 1:** Anthropometric characteristics of US data and Chinese populations

	US data (*n* = 11,844)				Chinese data (*n* = 51,229)				*P* value (NHANES vs Chinese)
	Total (*n* = 11,844)	non-NAFLD Group (*n* = 7571)	NAFLD Group (*n* = 4273)	*P* value	Total (*n* = 51,229)	non-NAFLD Group (*n* = 37,334)	NAFLD Group (*n* = 13,895)	*P* value	
Male gender, n(%)	5325(45.0%)	3332(44.0%)	1993(46.6%)	0.006	30,582(59.7%)	19,179(51.4%)	11,403(82.1%)	< 0.001	< 0.001
Age (years)	43.86 ± 16.08	42.18 ± 16.03	46.82 ± 15.75	< 0.001	43.49 ± 14.30	42.12 ± 14.48	47.18 ± 13.12	< 0.001	0.023
BMI (Kg/m^2^)	27.38 ± 5.90	26.16 ± 5.02	29.56 ± 6.67	< 0.001	23.08 ± 3.31	22.07 ± 2.87	25.82 ± 2.82	< 0.001	< 0.001
Diabetes, n(%)	896(7.6%)	354(4.7%)	542(12.7%)	< 0.001	3544(6.9%)	1540(4.1%)	2004(14.4%)	< 0.001	< 0.001
Hypertension, n(%)	2935(25.0%)	1600(21.3%)	1335(31.5%)	< 0.001	11,176(21.8%)	5821(15.6%)	5355(38.5%)	< 0.001	< 0.001
Blood glucose (mmol/l)	5.62 ± 2.09	5.35 ± 1.52	6.11 ± 2.77	< 0.001	5.36 ± 1.04	5.22 ± 0.83	5.74 ± 1.38	< 0.001	< 0.001
Mean arterial pressure (mmHg)	91.50 ± 28.34	89.97 ± 27.65	97.17 ± 30.10	< 0.001	92.03 ± 12.39	89.58 ± 11.84	98.61 ± 11.43	< 0.001	0.002
Diastolic pressure (mmHg)	75.29 ± 28.92	74.43 ± 31.34	76.83 ± 29.96	< 0.001	76.61 ± 11.33	74.39 ± 10.73	82.56 ± 10.75	< 0.001	< 0.001
Systolic pressure (mmHg)	123.91 ± 30.45	122.03 ± 31.75	1276.26 ± 27.67	< 0.001	122.88 ± 16.62	119.96 ± 16.06	130.71 ± 15.55	< 0.001	< 0.001

**Table 2:** Biochemical measurements of US data and Chinese populations

	US data (*n* = 11,844)				Chinese data (*n* = 51,229)				*P* value (NHANES vs Chinese)
	Total (*n* = 11,844)	non-NAFLD Group (*n* = 7571)	NAFLD Group (*n* = 4273)	*P* value	Total (*n* = 51,229)	non-NAFLD Group (*n* = 37,334)	NAFLD Group (*n* = 13,895)	*P* value	
Serum cholesterol (mmol/l)	5.29 ± 1.18	5.21 ± 1.15	5.42 ± 1.22	< 0.001	4.71 ± 0.94	4.58 ± 0.90	5.04 ± 0.98	< 0.001	< 0.001
Serum triglyceride (mmol/l)	1.65 ± 1.28	1.56 ± 1.21	2.00 ± 1.46	< 0.001	1.57 ± 1.42	1.26 ± 1.02	2.39 ± 1.92	< 0.001	< 0.001
Uric acid (umol/l)	313.86 ± 87.36	306.04 ± 82.63	342.51 ± 97.66	< 0.001	307.37 ± 92.74	287.9 ± 86.75	359.69 ± 87.96	< 0.001	< 0.001
NFS	−2.09 ± 1.63	−2.36 ± 1.50	−1.10 ± 1.71	< 0.001	−1.76 ± 1.24	−1.83 ± 1.22	−1.55 ± 1.25	< 0.001	< 0.001
FIB4	0.93 ± 0.65	0.82 ± 0.56	1.30 ± 0.81	< 0.001	1.32 ± 0.85	1.31 ± 0.88	1.33 ± 0.75	0.010	< 0.001
APRI	0.21 ± 0.19	0.21 ± 0.19	0.22 ± 0.20	0.002	0.34 ± 0.26	0.31 ± 0.26	0.38 ± 0.24	< 0.001	< 0.001

The prevalence of NAFLD was 27.12% in the Chinese population and 36.08% in the US population (*p* < 0.001). The NAFLD group had significantly higher age, BMI, blood glucose, blood pressure, cholesterol, and triglyceride level than the non-NAFLD group in both populations. The Chinese population had a higher eGFR level than the US population (91.48 ± 17.06 vs. 80.15 ± 18.65, *p* < 0.001). Moreover, the proportion of CKD, especially the proportion of CKD stage 3–5, was lower in the Chinese participants than the US participants (for CKD: 9.1% vs.21.5%; for CKD stage 3–5: 4.0% vs.13.5%, both *p* < 0.001).

### Renal function and NAFLD in different populations

As shown in Table 3, CKD was more prevalent in the NAFLD group than the non-NAFLD group in both populations (Chinese population: 12.3% vs. 8.0%, US population: 25.8% vs. 19.0%, both *p* < 0.001). However, there were some differences in renal function between these two groups in different populations. The mean serum creatinine levels were not statistically different between NAFLD group and non-NAFLD group in the US population (93.35 ± 25.88 vs. 93.62 ± 30.90, *p* = 0.636) while the eGFR were significantly lower in NAFLD group (78.45 ± 18.78 vs. 81.10 ± 18.51, *p* < 0.001) in the US population. In Chinese population, both the serum creatinine and eGFR showed statistically different between two groups. As for the CKD with different stages, late stages CKD (stages 3–5) consisted of 2/3 of CKD patients in the US population while less than half of CKD patients were in stages 3–5 among Chinese population.

**Table 3:** Comparison of the renal function index between US data and Chinese data

	US data (*n* = 11,844)				Chinese data (*n* = 51,229)				*P* value (NHANES vs. Chinese)
	Total (*n* = 11,844)	non-NAFLD Group (*n* = 7571)	NAFLD Group (*n* = 4273)	*P* value	Total (*n* = 51,229)	non-NAFLD Group (*n* = 37,334)	NAFLD Group (*n* = 13,895)	*P* value	
Serum creatinine (umol/l)	93.52 ± 29.19	93.62 ± 30.90	93.35 ± 25.88	0.636	82.92 ± 20.82	80.92 ± 21.70	88.29 ± 17.14	< 0.001	< 0.001
eGFR (ml/min/1.73^2^)	80.15 ± 18.65	81.10 ± 18.51	78.45 ± 18.78	< 0.001	91.48 ± 17.06	93.03 ± 17.31	87.32 ± 15.62	< 0.001	< 0.001
Proteinuria, n(%)	1305(11.0%)	714(9.4%)	591(13.8%)	< 0.001	3231(6.3%)	1977(5.3%)	1254(9.0%)	< 0.001	< 0.001
CKD, n(%)	2544(21.5%)	1441(19.0%)	1103(25.8%)	< 0.001	4685(9.1%)	2971(8.0%)	1714(12.3%)	< 0.001	< 0.001
CKD Categories, n(%)									
G0, n(%)	9300(78.5%)	6130(81.0%)	3170(74.2%)		46,544(90.9%)	34,363(92.0%)	12,181(87.7%)	< 0.001	< 0.001
G1–2, n(%)	958(8.1%)	521(6.9%)	434(10.2%)		2659(5.2%)	1616(4.4%)	1043(7.5%)	< 0.001	< 0.001
G3–5, n(%)	1586(13.5%)	917(12.1%)	669(15.7%)		2026(4.0%)	1355(3.6%)	671(4.8%)	< 0.001	< 0.001

### Associations between NAFLD and different stages of CKD

Table 4 shows the risk for CKD in NAFLD patients in different populations. In univariate logistic regression, NAFLD was significantly associated with CKD in both Chinese and US adults. In Chinese population, the NAFLD increased the risk of CKD but the strength of this affect attenuated with more confounding factors being adjusted: after adjusted for age, sex, BMI and histories of hypertension and diabetes, the OR dropped from 1.627 to 1.101. In the US population, the association between NAFLD and CKD was insignificant after adjusting for the same confounding factors (*P* > 0.05).

**Table 4:** Binary logistic regression analysis of NAFLD and CKD

Risk for CKD		US data			Chinese data		
		OR ^a^	95% CI	*P* value	OR^a^	95% CI	*P* value
All stages of CKD	Unadjusted	1.480	1.354–1.619	< 0.001	1.627	1.528–1.733	< 0.001
	Model1	1.071	0.964–1.191	0.203	1.181	1.094–1.274	< 0.001
	Model 2	0.993	0.891–1.107	0.902	1.101	1.019–1.189	0.015
CKD stages3–5	Unadjusted	1.347	1.210–1.500	< 0.001	1.347	1.226–1.481	< 0.001
	Model1	0.921	0.804–1.055	0.235	1.034	0.913–1.171	0.594
	Model 2	0.904	0.788–1.038	0.152	1.025	0.904–1.162	0.698
CKD stages 1–2	Unadjusted	1.520	1.331–1.737	< 0.001	1.794	1.655–1.944	< 0.001
	Model1	1.284	1.116–1.479	0.001	1.513	1.374–1.666	< 0.001
	Model 2	1.160	1.003–1.340	0.045	1.346	1.220–1.485	< 0.001

Model 1 Adjusted for age, sex, BMI. Model 2 Adjusted for age, sex, BMI, history of diabetes, history of hypertension

^a^Odds Ratios for associations between NAFLD and risk of CKD

To explore the possible explanation for this discrepancy between two countries, we performed a subgroup analysis by stratifying cases by the severity of CKD. As shown in Table 3, the US population had higher proportion of late stages CKD (CKD stages 3–5), so firstly, we evaluated the risk for late stages of CKD. The results showed that NAFLD was not significantly associated with late stage CKD in both Chinese population and the US population after adjustment for same metabolic factors (all P > 0.05). Then we excluded those with late stages CKD and calculated the risk of early stages of CKD in NAFLD patients. As shown in Table 4, NAFLD was an independent risk for early renal function decline in both populations after adjustment (OR 1.346–1.513 in Chinese population and 1.160–1.284 in US population, all *P* < 0.05).

## Discussion

NAFLD and CKD share similar pathological mechanisms; therefore they are speculated to have some links. This cross-sectional study analyzed the association between ultrasound-defined NAFLD and CKD in two health examination datasets from the US and China. The results of this study confirmed the higher prevalence of CKD among patients with NAFLD. Slightly difference was found between Chinese and US population in this study: in the Chinese population, NAFLD was significantly associated with increased risk of CKD after adjustment for metabolic factors, but in the US population, no significant association was found after adjustment for the same factors. However, when we excluded patients with advanced reduced renal function (eGFR< 60 ml/min/ 1.73 m^2^, CKD stages 3–5), NAFLD was significantly correlated with increased risk for early renal function decline in both populations.

The discrepancy between eastern and western population regarding the relationship between NAFLD and CKD has already been found by previous studies. Sirota et al. [15] found that NAFLD is not associated with the prevalence of CKD among US adults after adjusting for features of metabolic syndrome. While other studies from Asia reported a strong independent risk of CKD in ultrasonography-diagnosed NAFLD patients [16, 19, 20]. The meta-analysis also confirmed the difference between Asian and European populations [9]. The reason for this discrepancy has not been clarified since all the studies were not analyzed under same statistical condition. In this study, in order to compare the association of NAFLD and CKD in different populations, we creatively calculated the risks for CKD by adjustment for same confounders in different datasets. Consistent with previous studies, we confirmed the ethnic difference in the relationship between CKD and NAFLD in two population-based datasets.

The answer for the ethnic difference on the relationship between NAFLD and CKD might result from different proportion of severe renal dysfunction. The US population had a higher percentage of stages 3–5 CKD than the Chinese population. In fact, after we excluded those with late stages of CKD, NAFLD was strongly associated with early renal decline in both populations. Supporting our results, several prospective studies confirmed the influence of NALFD on the development of CKD, most of which were developed from the early stages [21–23]. But an important fact we should emphasize is that, compared with the early stage of CKD, the later stage of CKD is more complicated and severe. For example, increased synthesis and decreased clearance of triglycerides, extremely altered glucose homeostasis and uncontrollable blood pressure are more common among patients with late stages CKD [24–26]. Thus NAFLD might have an impact on renal function, yet it alone apparently not strong enough to contribute to the late stage function decline when severe renal decline is concerned. That explains why the association between late stages of CKD and NAFLD is not significant in both populations. When there are more late stages of CKD, such as the US population, the relationship of NALFD and CKD might attenuate during multivariate analysis.

To our best knowledge, this study is the first to compare the association between NAFLD and early renal function decline across different ethnicities. And we also answer the questions of conflicting conclusion from different countries from some aspects. The results of our study suggest that NAFLD plays a more important role in mild renal dysfunction, which providing more evidences for the hypothesis that pre-existing NAFLD is an independent risk factor for the development of renal injury.

There are several limitations in this study that deserve a mention. Firstly, the diagnosis of CKD usually requires the presence of an abnormality of kidney function or kidney structure for more than 3 months; however, it is difficult to perform a second examination in such a large population in a population-based study. We had to admit that the diagnostic criteria used in this study, which has also been used by several similar researches [15, 27, 28], might overestimate the prevalence of CKD. Given the cross-sectional design of this study, we are unable to draw conclusions about the causality of the relationship between NAFLD and early renal function decline. Secondly, we have not use cystatin C to define eGFR, which is less affected than creatinine by muscle mass and more accurate for different ethnicities. Thirdly, there is a huge difference in period of database between the US (1984–1994) and Chinese (2010) populations, therefore, the discrepancy in the diet and lifestyle might be a major concern. It is widely accepted that patients with CKD should limit the intake of certain foods to reduce the accumulation of unexcreted metabolic products and also to protect against hypertension, proteinuria and other health problems. In fact, the diet and lifestyle in China are closer to that in western country since twenty-first century. According to a previous report, China’s food consumption patterns and dietary behaviors changed dramatically between 1991 and 2011. The diet has shifted from macronutrient composition toward fats, and protein and sodium intakes [29]. Another study also demonstrated that the structure of the Chinese diet has been shifting away from the traditional diet toward high-fat, low-carbohydrate and low-fiber diets, and nutrients intakes in Chinese people have been changing even worse than those in American people [30]. Therefore, the heterogeneous database might partially influence the results, but the impact is not as larger as expected.

## Conclusions

Our findings demonstrated a significant positive association between the presence of NAFLD and early stage of CKD in both the US and Chinese adults. For the late stages of CKD, NAFLD might not be strong enough to be an independent contributor.

## Data Availability

The NHANES III database is a public database which is available at https://www.cdc.gov/nchs/nhanes/about_nhanes.htm. The Chinese database would be available on request.

## Abbreviations

NAFLD: Non-alcoholic fatty liver disease
CKD: Chronic kidney disease
NHANES III: The Third American National Health and Nutrition Examination Survey
BMI: Body mass index
MAP: Mean arterial pressure
eGFR: Estimated glomerular filtration rate
KIDGO: Kidney Disease: Improving Global Outcome

## References

[1] Araujo AR (2018). Global epidemiology of non-alcoholic fatty liver disease/non-alcoholic steatohepatitis: what we need in the future. Liver Int.

[2] Han E, Lee YH (2017). Non-alcoholic fatty liver disease: the emerging burden in Cardiometabolic and renal diseases. Diabetes Metab J.

[3] Wu D (2019). Nonalcoholic fatty liver disease aggravated the severity of acute pancreatitis in patients. Biomed Res Int.

[4] Sookoian S, Pirola CJ (2019). Review article: shared disease mechanisms between non-alcoholic fatty liver disease and metabolic syndrome – translating knowledge from systems biology to the bedside. Aliment Pharmacol Ther.

[5] Abenavoli L (2016). Metabolic aspects of adult patients with nonalcoholic fatty liver disease. World J Gastroenterol.

[6] Byrne CD, Targher G (2020). NAFLD as a driver of chronic kidney disease. J Hepatol.

[7] Kumela Goro K (2019). Patient Awareness, Prevalence, and Risk Factors of Chronic Kidney Disease among Diabetes Mellitus and Hypertensive Patients at Jimma University Medical Center, Ethiopia. Biomed Res Int.

[8] Targher G, Chonchol MB, Byrne CD (2014). CKD and nonalcoholic fatty liver disease. Am J Kidney Dis.

[9] Mantovani A (2018). Nonalcoholic fatty liver disease increases risk of incident chronic kidney disease: a systematic review and meta-analysis. Metabolism.

[10] Mantovani A (2020). PNPLA3 I148M gene variant and chronic kidney disease in type 2 diabetic patients with NAFLD: clinical and experimental findings. Liver Int.

[11] Sun DQ (2020). PNPLA3 rs738409 is associated with renal glomerular and tubular injury in NAFLD patients with persistently normal ALT levels. Liver Int.

[12] Wilechansky RM (2019). Relations of liver fat with prevalent and incident chronic kidney disease in the Framingham heart study: a secondary analysis. Liver Int.

[13] Singal AK (2016). Nonalcoholic Steatohepatitis is the Most rapidly growing indication for simultaneous liver kidney transplantation in the United States. Transplantation.

[14] Houlihan DD (2011). Renal function in patients undergoing transplantation for nonalcoholic steatohepatitis cirrhosis: time to reconsider immunosuppression regimens?. Liver Transpl.

[15] Sirota JC (2012). Association between nonalcoholic liver disease and chronic kidney disease: an ultrasound analysis from NHANES 1988-1994. Am J Nephrol.

[16] Yun KE (2009). Elevated alanine aminotransferase levels predict mortality from cardiovascular disease and diabetes in Koreans. Atherosclerosis.

[17] Levey AS (2009). A new equation to estimate glomerular filtration rate. Ann Intern Med.

[18] Inker LA (2014). KDOQI US commentary on the 2012 KDIGO clinical practice guideline for the evaluation and management of CKD. Am J Kidney Dis.

[19] Li G (2012). Nonalcoholic fatty liver disease associated with impairment of kidney function in nondiabetes population. Biochem Med (Zagreb).

[20] Wang L (2019). Ultrasound-diagnosed nonalcoholic fatty liver disease independently predicts a higher risk of developing diabetes mellitus in nonoverweight individuals. Acad Radiol.

[21] Arase Y (2011). The development of chronic kidney disease in Japanese patients with non-alcoholic fatty liver disease. Intern Med.

[22] Chang Y (2008). Nonalcoholic fatty liver disease predicts chronic kidney disease in nonhypertensive and nondiabetic Korean men. Metabolism.

[23] Targher G (2008). Increased risk of CKD among type 2 diabetics with nonalcoholic fatty liver disease. J Am Soc Nephrol.

[24] Chmielewski M (2008). Lipid disorders in chronic kidney disease: reverse epidemiology and therapeutic approach. J Nephrol.

[25] Plantinga LC (2009). Blood pressure control among persons without and with chronic kidney disease: US trends and risk factors 1999–2006. Hypertension.

[26] Moradi H, Vaziri ND (2018). Molecular mechanisms of disorders of lipid metabolism in chronic kidney disease. Front Biosci (Landmark Ed).

[27] Li Y (2014). Association between non-alcoholic fatty liver disease and chronic kidney disease in population with prediabetes or diabetes. Int Urol Nephrol.

[28] Targher G (2010). Relationship between kidney function and liver histology in subjects with nonalcoholic steatohepatitis. Clin J Am Soc Nephrol.

[29] Zhai FY (2014). Dynamics of the Chinese diet and the role of urbanicity, 1991–2011. Obes Rev.

[30] Zhang R (2015). The difference in nutrient intakes between Chinese and Mediterranean*,* Japanese and American Diets. Nutrients.

